# Ethnobotanical uses, phytochemistry and biological activity of the genus *Euclea:* A review

**DOI:** 10.3389/fphar.2023.1170145

**Published:** 2023-04-19

**Authors:** Abebe Dagne Taye, Gizachew Kassahun Bizuneh, Asmamaw Emagn Kasahun

**Affiliations:** ^1^ Department of Pharmacy, College of Health Sciences, Debre Markos University, Debre Markos, Ethiopia; ^2^ Department of Pharmacognosy, School of Pharmacy, College of Medicine and Health Sciences, University of Gondar, Gondar, Ethiopia; ^3^ Department of Pharmaceutics, School of Pharmacy, College of Medicine and Health Sciences, University of Gondar, Gondar, Ethiopia

**Keywords:** *Euclea*, naphtoquinones, phytochemistry, ethnobotanical use, pharmacological activity

## Abstract

*Euclea (*Ebenaceae*)* is a genus of flowering shrubs and trees widely distributed in Africa, the Comoro Islands, and Arabia. This review aimed to evaluate the ethnobotanical uses, phytochemistry, and biological activities of the genus *Euclea* on available research reports. This was achieved through PubMed, Medline, Google Scholar, Science Direct, Taylor and Francis Online, Wiley Online Library which provides access to scientific and medical research. The extensive literature survey revealed that plants that belong to this genus are used as folkloric medicine for the treatment of diabetes mellitus, toothache, diarrhea, cancer, malaria, leprosy, and genital and oral diseases in the case of HIV/AIDS-related diseases. To date, more than 40 secondary metabolites have been isolated and identified from these plants, especially from *E natalensis* and *E. divinorum.* Among these, naphthoquinones, terpenes, and flavonoids are potential secondary metabolites with profound biological activities. *Euclea* plant extracts and their bioactive compounds possess outstanding pharmacological properties, especially antimalarial, antidiabetic, anticancer, antimicrobial, and antioxidant properties.

## Introduction

The word “*Euclea*” comes from a Greek word “eukleia”, “eu” meaning “good”, and “kleios”meaning report (Maroyi, 2017). The genus *Euclea* belongs to the family Ebenaceae and is composed of 16 accepted species (Dhayalan et al., 2015; Botha, 2016).

The genus *Euclea* is distributed in the tropical and subtropical regions of the world. However, it is most abundant in Eastern and Southern Africa (Mebe et al., 1998) and South-East Asia (Botha, 2016). *Euclea divinorum* is distributed in Botswana, South Africa, Namibia, Swaziland, Zimbabwe, Tanzania, Uganda (Shumba, 2018), Sudan, Kenya, and Ethiopia (Woldemedhin et al., 2017). *Euclea natalensis* is widely found along the eastern coast of southern Africa (Johanna, 2007). *Euclea latideus* is well presented in the lowlands of the tropical and to a lesser extent, in subtropical regions of the world (Philip et al., 2018). A versatile medicinal plant in Ethiopia from this genus is *Euclea divinorum.* Traditionally it is used for the treatment of skin inflammation, scabies, cancer, hepatitis, urinary inconsistency, chest pain, pneumonia, gonorrhea, constipation, edema, abdominal and chest pain (Feyissa et al., 2013; Woldemedhin et al., 2017; Mekonnen et al., 2018).

### Botanical profile and taxonomy of *Euclea*

**FIGURE 1:**
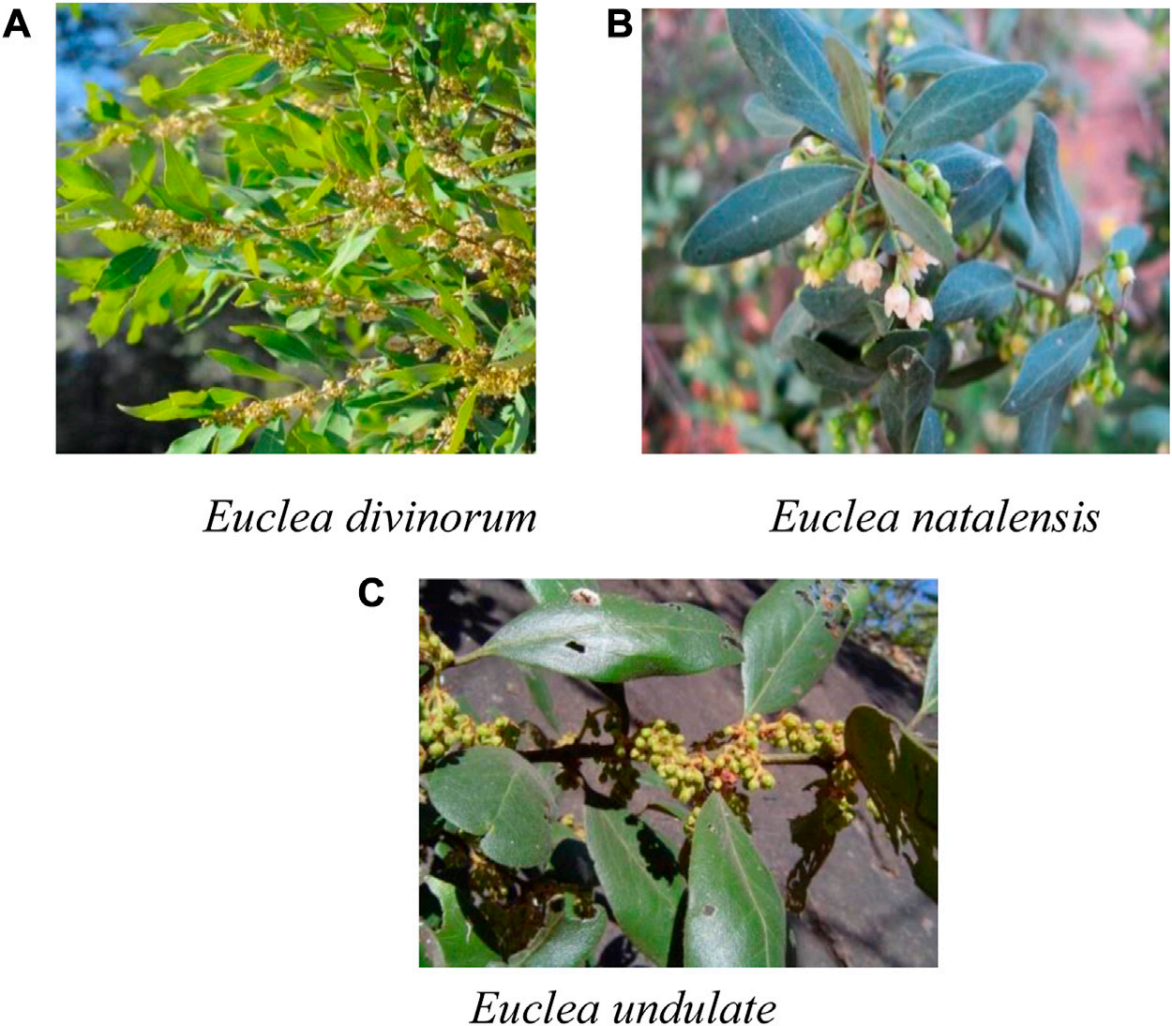
**(A)** *Euclea divinorum* **(B)** *Euclea natalensis* **(C)** *Euclea undulate*.

Most of the plants are trees, shrubs, and sub-shrubs, usually evergreen with alternate, opposite to sub-opposite, or in pseudo-whorls and diamond leaved (Figure 1A). Inflorescences: dioecious, axillary, or less frequently in branched pseudo-racemes, or flowers occasionally solitary (Figure 1B). Calyx: 4-5-lobed, usually polysepalous, not accrescent on fruits. Corolla: urceolate to subglobose, 5 - 8-lobed or campanulate and deeply 4-5-lobed. Stamens: 10-30; anthers dehiscing by large ellipsoidal apical pores, hairy or glabrous, oblong or lanceolate, 2-celled; filaments short, usually slender and glabrous. Staminodes: usually absent, glabrous; styles 2 (or 1, bifid), usually glabrous; stigmas bifid at apex. Ovary: ovoid or globular, hairy or glabrous, usually 4-celled; ovules 4, pendulous. Fruit: usually globose, 1-seeded berry (Halim et al., 2014), edible, spherical and one-seeded berries (Figure 1C) (Dhayalan et al., 2015). Many members of this genus are traditionally used to treat different diseases. Some are scientifically investigated for various biological activities and phytoconstituents. Previously, reviews that focus on single species, *E undulata* Thunb (Maroyi, 2017) and *E. divinorum* Hiern (Omara et al., 2020) have been conducted. To the authors’ knowledge, no study reviewed the ethnopharmacological use, phytochemistry, and biological activities of the whole genus.

### Methodology

**FIGURE 2:**
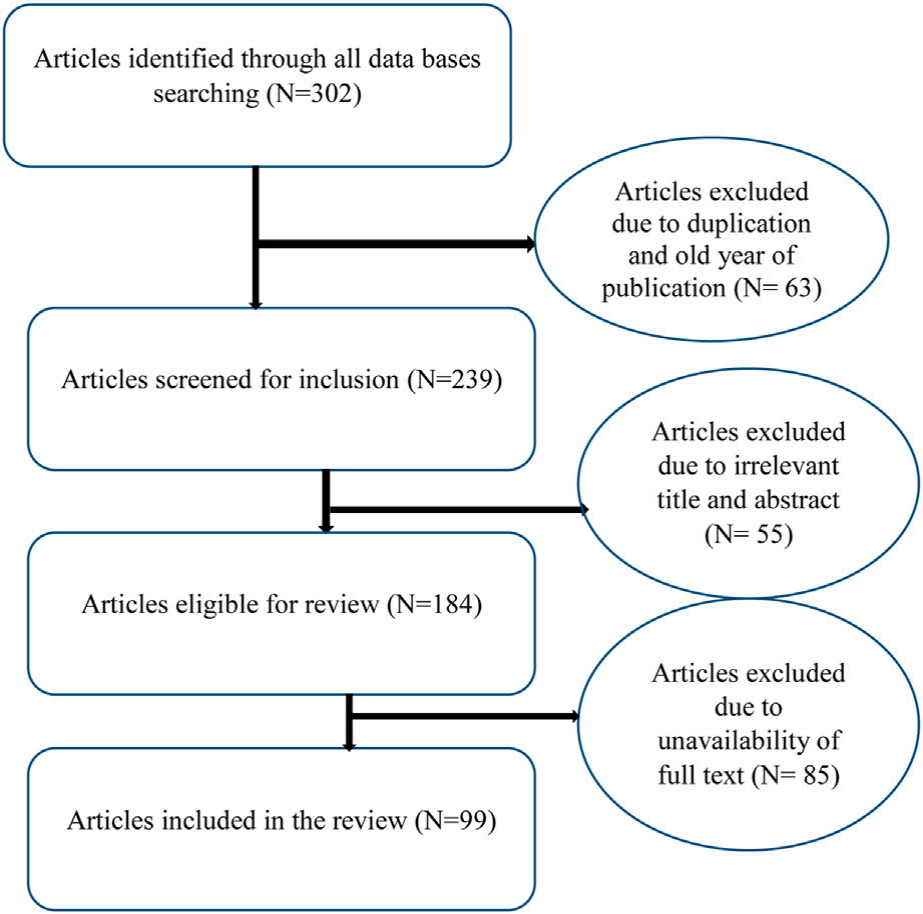
Flowchart of reviews included and excluded.

This review aims to critically evaluate available research reports on the genus and systematically organize and present the results. The review summarizes the existing knowledge on the ethnobotanical use, phytochemistry, and pharmacological activity of species belonging to the genus *Euclea* to bring the reader up to date with the current literature. Articles on the species of the genus *Euclea* that reported ethnobotanical uses, biological activities, and isolation and identification of compounds were included. It is attempted to include articles published from 1975–2023 while some articles published before 1975 were also included by considering their importance. In this review articles where the full text was not available in the database or even after contacting the author by email were excluded (Figure 2).

This review excluded unpublished results and publications unavailable online, articles written in languages other than English, and articles whose titles and abstracts did not contain the search terms. Chemical structures of only isolated and characterized compounds were provided, while structures of compounds identified from essential oils and other chemical analyses were not. Different databases, including PubMed, Google Scholar, Scopus, and Medline, were employed to search literature using “keywords such as “*Euclea*”, “ethnobotanical use”, “phytochemistry”, and “pharmacological activity” dated up to December 2023.”

### Ethno pharmacological uses

Ethnomedicinal claims on the genus *Euclea* to treat several ailments are illustrated in Table 1. The genus *Euclea* is used to treat hypnosis, toothache, headache (Bapela et al., 2008; Babula et al., 2009), chest complaints, bronchitis, pleurisy, chronic asthma, urinary tract infections, and venereal diseases (Lall & Meyer, 2000; Lall and Meyer, 2001; Weigenand et al., 2004; Kooy et al., 2006; Johanna, 2007; Bapela et al., 2008). An infusion of the roots of *E. ceispa* possesses antiepileptic activity (Dhayalan et al., 2015). The root bark of *E. undulata* is reported to be used for the management of body pains, diabetes, headache, and toothache while an infusion of its leaves is used for stomach problems or diarrhea, and leaf decoction for tonsillitis (Deutschländer M. et al., 2009; Dhayalan et al., 2015; Maroyi, 2017). This plant is a folk medicine for diabetes in the Venda area, Limpopo Province (Deutschländer M. S. et al., 2009; Babiaka et al., 2015; Maroyi, 2017). In the Western Cape, the root infusion of *E*. *undulata* is used as enemata or as an ingredient of inembe (herbal medication regularly taken during pregnancy to ensure trouble-free confinement). Emesis or purgation is induced with root preparations (Deutschländer M. et al., 2009).

**TABLE 1:** Traditional uses of members of the genus *Euclea*.

Species	Part used	Indication	Country	References
*E. crispa*	R	cough	South Africa	Schmidt et al. (2002)
	R	constipation	South Africa	Chinsamy and Koitsiwe (2016)
			Swaziland	Long (2005)
	B, F, L, R	Diabetes	South Africa, Swaziland, Zimbabwe	Palmer and Pitman (1972) Chinsamy and Koitsiwe, 2016; Schmidt et al., 2002; Masoga, 2020
	L	Dysmenorrhoea	South Africa	Steenkamp (2003)
	R	convulsions and epilepsy	Zimbabwe	Stafford et al. (2008)
*E. coriacea*	B	Constipation, Stomach pains	South africa	Kose et al. (2015)
	R, L and S	Gonorrhea	Lesotho	Mugomeri et al., 2014
*E. divinorum*	R	HIV/AIDS-related diseases	Zambia	Chinsembu (2016)
	R	urine retention	Ethiopia	Woldemedhin et al. (2017)
	L	malaria, leprosy, gonorrhea, syphilis and tapeworm	Ethiopia	Geyid et al., 2005; Nanyingi et al., 2008
	R	induce labour	Kenya	Kaingu et al. (2012)
	F	abdominal upsets, skin, kidney and respiratory disorders	Kenya	Kigen et al. (2017)
	R	convulsions	Zimbabwe	Sobiecki (2002)
	R	schistosomiasis	South African	Sparg et al. (2000)
*E. kellau*	B	splenic pain	Tanzania	Orzalesi et al. (1970)
	R	Ancylostomiasis	Tanzania	Orzalesi et al. (1970)
	L	snake-bite		Orzalesi et al. (1970)
*E. natalensis*	R	Diabetes	South Africa; Kenya	Deutschländer M. S. et al., 2009 *;* Keter and Mutiso, 2012
	R	Epilepsy	South Africa	Van Wyk and Gericke (2000)
	L	Antidote for poisoning, snake bite	Malawi	Morris and Msonthi (1996)
	B	Prostate cancer	Uganda	Omara et al. (2020)
	R	Leprosy, syphilis, toothache	South Africa	Oosthuizen and Lall (2020)
	Sh and B	chest pains, bronchitis, pleurisy, and asthma	South Africa	Oosthuizen and Lall (2020)
	R	Herpes simplex virus 1	South Africa	Lall et al. (2005a)
		Tuberculosis	South Africa	Lall and Meyer (2001)
	B	schistosomiasis	South Africa	Sparg et al. (2000)
*E. racemosa*	R	Warts of the rectum, Constipation	Ethiopia	D’avigdor et al., 2014; Ayele et al., 2023
	RB	Cancer	Ethiopia	Wube et al. (2005)
	RB	toothache and malaria	Uganda	Neuwinger (2000)
	R	cancer, abdominal pain and convulsive dysmenorrhoea	Tanzania	Neuwinger (2000)
	RB, L, S, Br	Diabetes	Kenya	Keter and Mutiso (2012)
*E. schimper*	L	gonorrhea, eczema and constipation, snake biting, scabies, leprosy, Tinea capitis, acne, warts, rheumatic pain and elephantiasis	Ethiopia	Gelahun, 1989; Abebe and Ayehu, 1993; Mekonnen et al., 2018
	R	febrile disease (fever, headache and sweating)	Ethiopia	Giday et al. (2003)
	B	Dysmenorrhoea	South Africa	Steenkamp (2003)
*E. undulata*	RB	Diabetes	South Africa	Deutschländer M. et al., 2009 *;* Deutschlander, 2010
	B, R	Toothache	Botswana	Palmer and Pitman, 1972; Motlhanka and Nthoiwa, 2013
	L	Diarrhoea	South Africa	Deutschländer M. S. et al. (2009)
	S	Chewing stick	Zimbabwe	Joshua et al., 2013

AP, aerial part; B, bark; Br, Branch; F, flower; Fr, Fruit, L, leaf; R, root; RB, root bark; Sh, shoot; S, stem; Sd, Seed; WP, whole plant.

The Zulu people use *E. natalensis* as a purgative (Lall and Meyer, 2001; Weigenand et al., 2004) and for abdominal complaints in the form of infusion (Deutschländer M. S. et al., 2009; Deutschländer, 2010). Its charred and powdered root is used treat leprosy, urinary tract infections, venereal diseases, dysmenorrhea, and ancylostomiasis among Shangaan people (Lall & Meyer, 2000; Lall and Meyer, 2001); Kooy et al., 2006*;* Deutschländer, 2010) while its root bark infusions for sores and wounds in South Africa (Lall and Meyer, 2001). Within the Tonga people, the same part of this plant exhibits toothache and headache relief (Deutschländer M. et al., 2009; Babiaka et al., 2015; Dhayalan et al., 2015).

In Swaziland, the stem bark decoction of *E. divinorum* is a folk medicine for constipation (Amusan et al., 2007). The root bark is used for diarrhea, convulsions, cancer, and skin diseases (Mebe et al., 1998; Babiaka et al., 2015). In Kenya, the root of this plant is a remedy for chest pain, pneumonia, and internal body swelling (Woldemedhin et al., 2017). In Ethiopia, the roots and leaves of this plant are used for treating urinary retention, malaria, leprosy, gonorrhea, syphilis, and tapeworm (Feyissa et al., 2013; Woldemedhin et al., 2017). *E. schimperi* is traditionally prescribed for managing wounds, teeth infection, eye disorder, headache, gonorrhea, eczema, skin disorder, snake biting, scabies, leprosy, and elephantiasis in Ethiopia (Mekonnen et al., 2018).

### Phytochemistry

Euclea is a good source of naphthoquinones, pentacyclic triterpenes (Dagne et al., 1993; Joubert et al., 2006; Kwon et al., 2011; Dhayalan et al., 2015), flavonoids, naphthols (Dagne et al., 1993) and diosindigo (Dhayalan et al., 2015). Members of the genus *Euclea* contain primarily naphtoquinones and the root/root bark of the plant is the main source of the naphtoquinones. Phytochemical screening revealed that the leaf of E. schimperi contains saponins, terpenoids, tannins, steroids, polyphenols, and flavonoids after extraction with methanol and chloroform (Mekonnen et al., 2018). Aqueous and 80% methanol root extract of *E. divinorum* had shown to contain saponins, flavonoids, glycosides, steroids, tannins, and terpenoids (Woldemedhin et al., 2017; Al-fatimi, 2019) but alkaloids and anthraquinones were absent (Woldemedhin et al., 2017). On the other hand, the root bark of this plant produces alkaloids, terpenoids, flavonoids, tannins, and saponins (Shumba, 2018). Methanol leaf and stem extracts of E. undulata contained alkaloids, diterpenes, glycosides, phytosterols, reducing sugars, saponins, and tannins (Maroyi, 2017). Essential oils, saponins, terpenoid derivatives, alkaloids, and flavonoids are the constituents of *E. crispa* subsp. crispa (Kwon et al., 2011).

### Naphthoquinone

Quinones are one of the plant-derived secondary metabolites. Based on the number of benzene rings in the structural fused and skeleton, they are mainly classified as naphthoquinone, phenanthrenequinone, anthraquinone, and benzoquinone (Demir, 2020). Naphthoquinones are phenolic compounds derived from naphthalene occurring in plants (common) and fungi (Mbaveng & Kuete, 2014; Botha, 2016). They were mainly detected from the root barks of the genus *Euclea* (Khan, 1985). Naphthoquinone isolated from the genus *Euclea* is presented in Table 2.

**TABLE 2:** Naphthoquinones isolated from Euclea species.

No.	Name of the compound	Species	Plant part	Structure	References
1.	2-methylnaphthazarin	*E. pseudebenus*	R	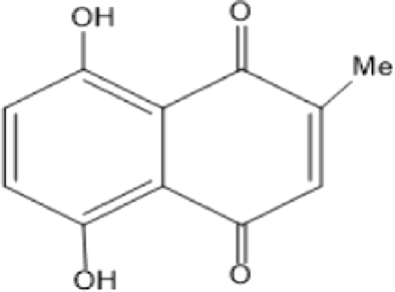	Ferreira et al., 1973; Dhayalan et al., 2015
2.	7-methyl-juglone	*E. undulata*	R	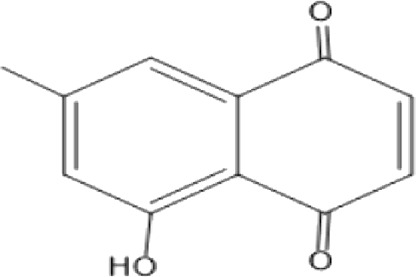	Deutschländer M. et al., 2009; Mbaveng & Kuete, 2014; Botha, 2016
		3*E.natalensis*	R		Mbaveng & Kuete, 2014; Deutschländer M. S. et al., 2009; Babula et al., 2009
		4*E. natalensis*	S, R and Sd		Lall et al., 2005b; Joubert et al., 2006; Johanna, 2007*;* Bapela et al., 2008; Babiaka et al., 2015; Kooy et al., 2006
		5*E. divinorum*	Us		Mebe *et a*l., 1998; Babula et al., 2009
		6*E. divinorum*	R		Al-fatimi (2019)
		7*E. pseudebenus*	R		Ferreira et al. (1973)
		8*E. racemosa* ssp. *schimperi*	R		Wube et al. (2005)
		9*E. lanceolata*	RB		Ferreira et al. (1974)
3.	8,8’-dihyd roxy-4,4’-dimethoxy-6, 6’-dimethyl-2,2’- binaphthyl-l,l’- quinone	*E. lanceolata*	RB	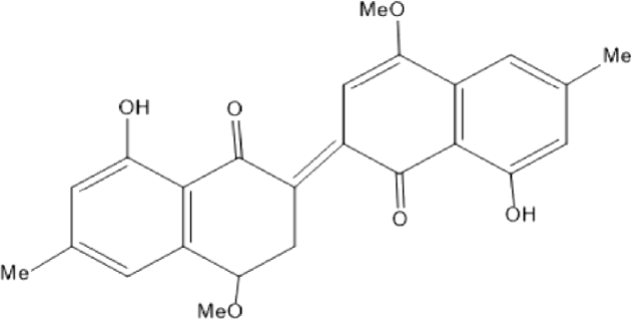	Ferreira et al. (1974)
4.	8’-hydroxydiospyrin	*E. lanceolata*	RB	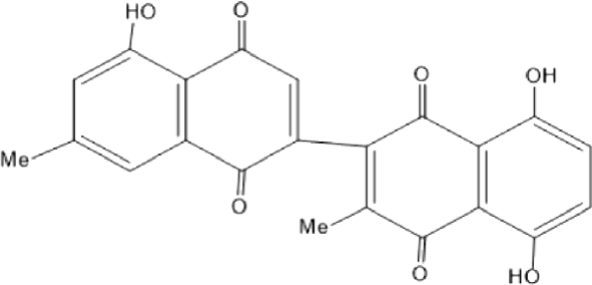	Ferreira et al. (1974)
5.	biramentaceone	*E. pseudebenus*	R	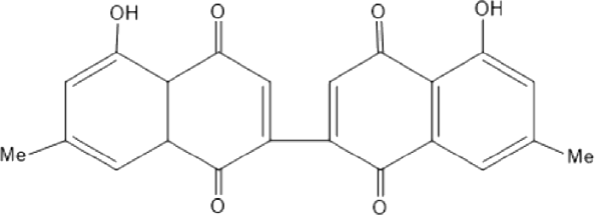	Ferreira et al. (1973)
6.	diospyrin	*E. undulata*	R	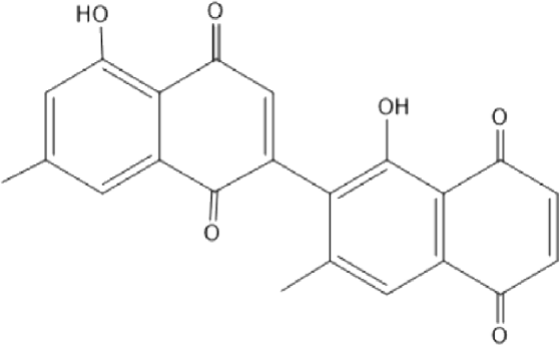	Deutschländer M. et al. (2009)
		*E. crispa var crispa*	R,Fr		Botha (2016)
		*E. undulata*	Fr		Botha (2016)
		*E. natalensis*	S, R and Sd		Lall et al., 2005a; Joubert et al., 2006; Johanna, 2007*;* Bapela et al., 2008; Babiaka et al., 2015
		*E. pseudebenus*	Us		Dhayalan et al. (2015)
		*E. divinorum*	R		Al-fatimi (2019)
		*E. natalensis*	Us		Deutschländer M. S. et al. (2009)
7.	eucleanal	*E. divinorum*	L	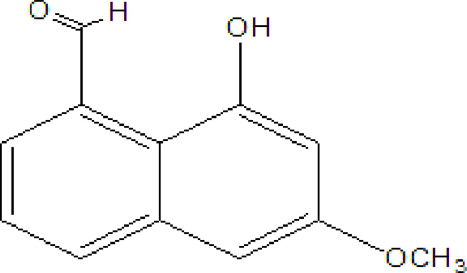	Ng’ang’a et al., 2012
8.	eucleanal B	*E. divinorum*	L	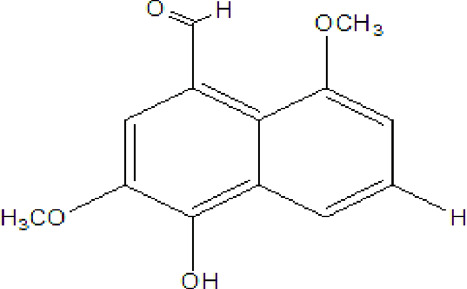	Mwihaki Ng’ang’a et al., 2012
9.	eucleanal A	*E. divinorum*	L	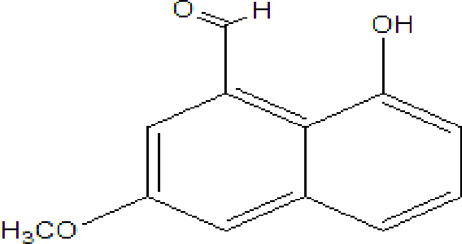	Mwihaki Ng’ang’a et al., 2012
10.	euclein (3, 6’-dimer of 7-methyljuglone.)	*E.pseudebenus*	R	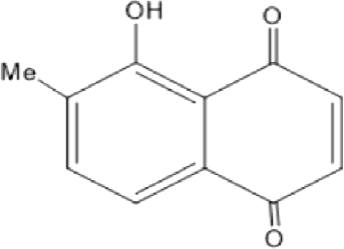	Ferreira et al. (1973)
11.	isodiospyrin	*E. undulata*	Fr	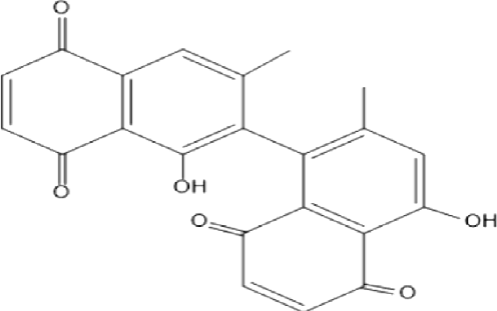	Deutschländer M. et al., 2009; Botha, 2016
		*E. crispa*	Fr		Botha (2016)
		*E. natalensis*	Us		Kooy et al., 2006; Wube et al., 2005
		*E. racemosa* ssp. *schimperi*	R		Wube et al., 2005; Al-fatimi, 2019
**12**.	mamegakinone	*E. natalensis*	Us	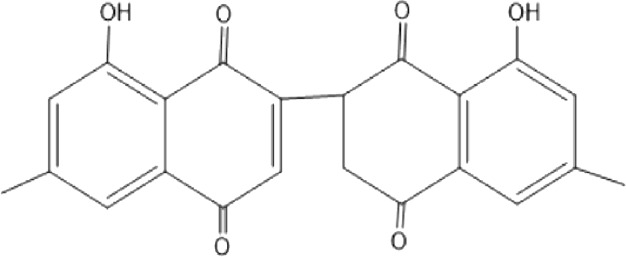	Kooy et al., 2006
		*E. natalensis*	Us		Deutschländer M. S. et al. (2009)
		*E. pseudebenus*	R		Ferreira *et al.*, 197
		*E. racemosa* ssp. *schimperi*	R		Wube et al. (2005)
		*E. natalensis, E. cripsa,* and *E. schimperi*	Us		Dhayalan et al. (2015)
		*E. divinorum*	R		Al-fatimi (2019)
		*E. lanceolata*	RB		Ferreira et al. (1974)
13.	natalonone	*E. crispa* subsp. *Crispa*	Us	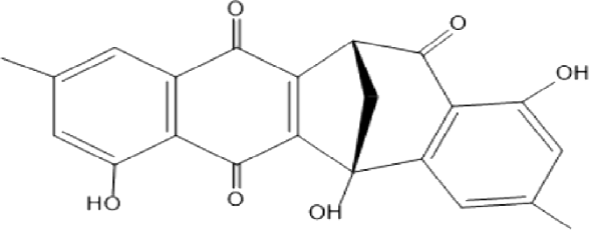	Kwon et al. (2011)
14.	neodiospyrin	*E. natalensis*	S, R and Sd	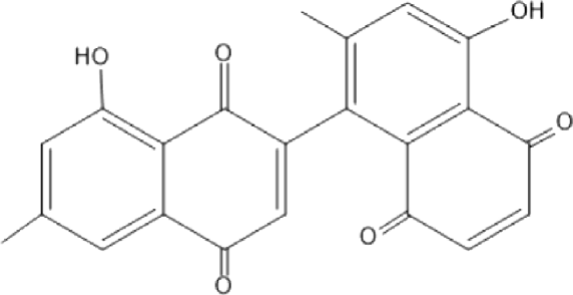	Joubert et al., 2006; Johanna, 2007*;* Bapela et al., 2008; Babiaka et al., 2015
		15*E. racemosa* ssp. *schimperi*	R		Wube et al. (2005)
15.	octahydroeuclein	*E. natalensis*	RB	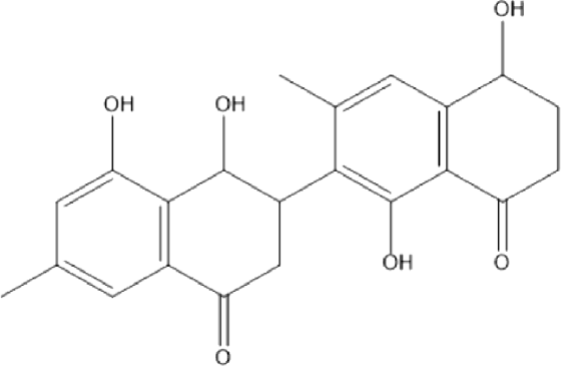	Weigenand et al., 2004; Lall et al., 2006
16.	shinanolone	*E. natalensis*	RB	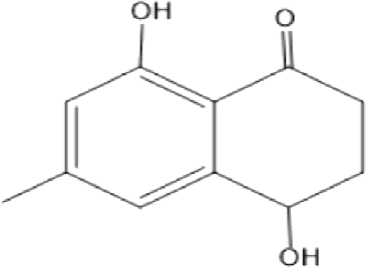	Weigenand et al., 2004; Kooy et al., 2006; Lall et al., 2006
		18*E. natalensis*	S, R and Sd		Joubert et al., 2006; Johanna, 2007*;* Bapela et al., 2008
		19*E. divinorum*	Us		Mebe et al. (1998)
		20*E.racemosa* ssp. *schimperi*	R		Wube et al. (2005)
		21*E. natalensis*	Us		Kooy et al., 2006

B, bark; F, flower; Fr, Fruit; R, root; RB, root bark; S, shoot; Sd, Seed; Us, Unspecified.

### Flavonoids

Flavonoids are phenolic compounds having two benzene rings linked through a heterocyclic pyrane ring (Shumba, 2018). Quercetin, kaempferol (Al-fatimi, 2019), new aromadendrin-3-O-β-L-arabinopyranoside (**17**), and known flavonoids such as catechin (Dagne et al., 1993; Mebe et al., 1998), myricetin-3-O-α-L–rhamnopyranoside (**21**) and quercetin-3-O-α-L-rhamnopyranoside (**22**) were isolated from the extract of ethanol aerial part of *E. divinorum* (Dagne et al., 1993), (Table 3). Acetone leaves extract of *E. racemosa* ssp. *Schimperi* yields quercetrin**,** myricitrin**,** myricetin-3-O-arabinopyranoside (**20**) and rutin (**23**), (Asres et al., 2006).

**TABLE 3:** Flavonoids isolated from Euclea species.

No.	Name of the compound	Species	Plant part	Structure	References
**17**	aromadendrin-3-O-β-L arabinopyranoside	*E. divinorum*	A	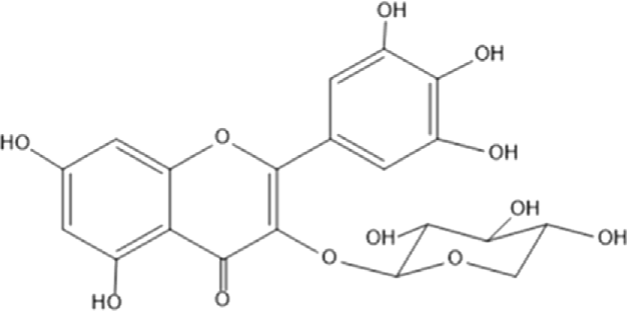	Dagne et al., 1993
**18**	catechin	*E. divinorum*	RB	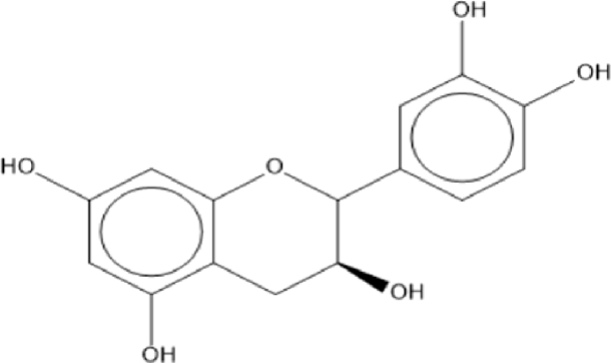	Babiaka et al. (2015)
**19**	epicatechin	*E. undulate*	RB	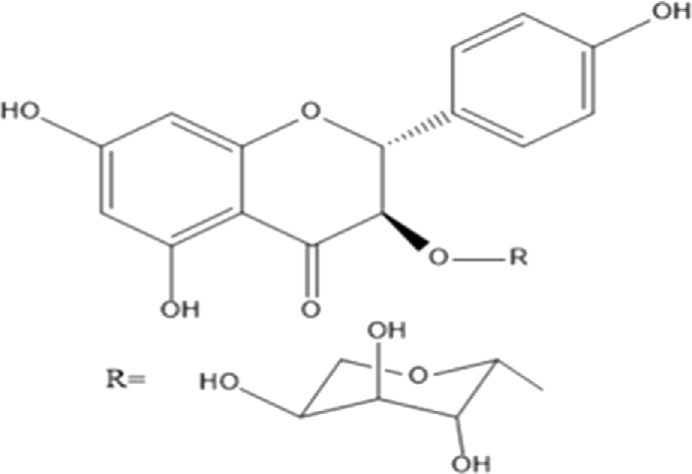	Deutschländer, 2010; Babiaka et al., 2015; Botha, 2016
**20**	myricetin-3-O-arabinopyranoside	*E. racemosa*	L	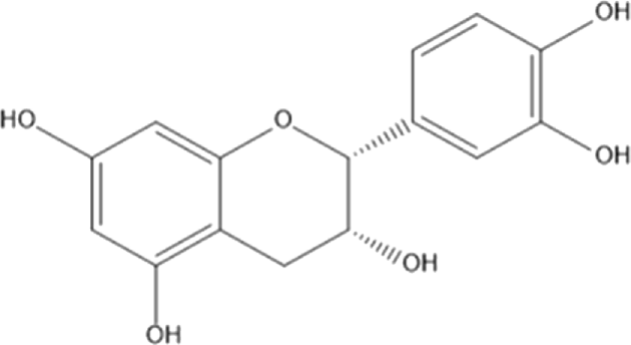	Asres et al. (2006)
**21**	myricetin-3-O-α-L –rhamnopyranoside	*E. divinorum*	A	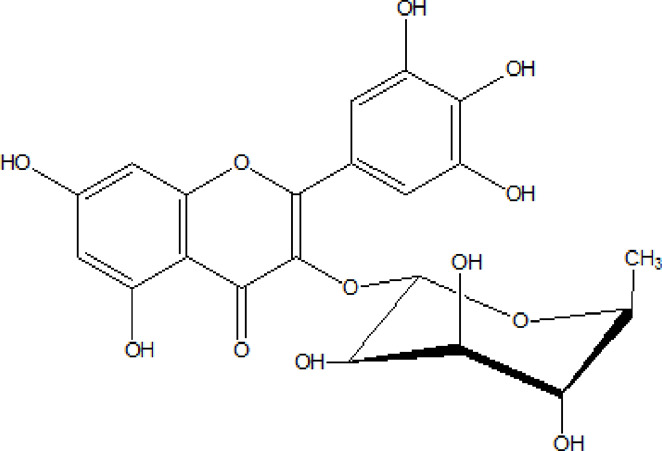	Dagne et al., 1993
**22**	quercetin-3-O-α-L-rhamnopyranoside	*E. divinorum*	A	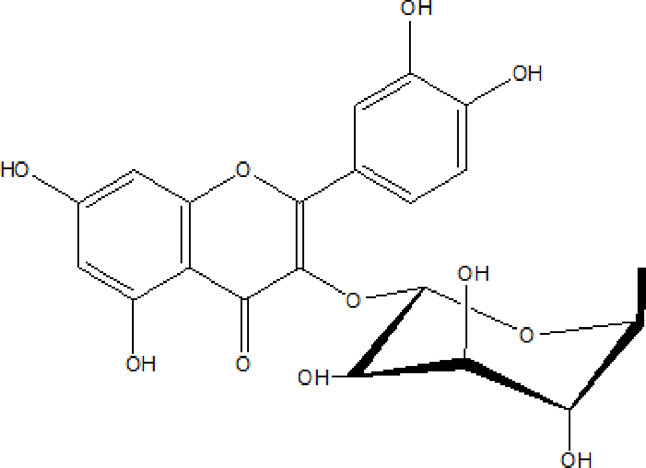	Dagne et al., 1993
**23**	rutin	*E. racemosa*	L	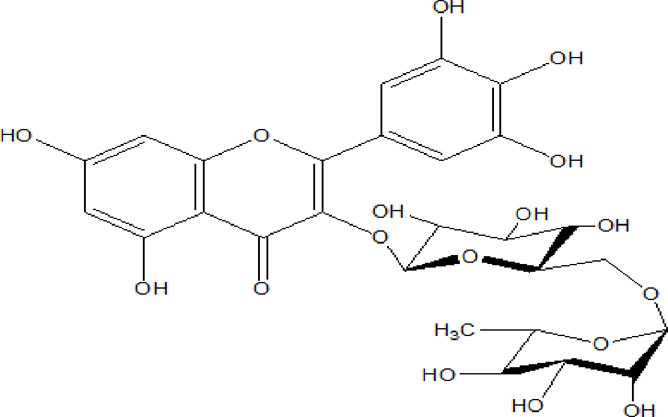	Asres et al. (2006)

A, arial; RB, root bark; L, leaf.

HPLC detects large amounts of myricitrin and small amounts of isoquercitrin and quercitrin in *E. schimperi* (Mueller-harvey et al., 1987)*.* Root bark extracts of *E. undulata* (acetone) (Deutschländer, 2010; Babiaka et al., 2015; Botha, 2016)*, E. divinorum* (chloroform) and E. *undulata* (acetone) resulted in the isolation of epicatechin (**19**) and catechin (**18)** respectively (Babiaka et al., 2015). Hyperoside, quercitrin, epicatechin, catechins and gallocatechin were isolated from the leaves of *E. crispa subsp. Crispa* (Rademana et al., 2019).

### Terpenoids

Triterpenes are a group of natural products, derived from isoprene units. In nature, triterpenoids are often existed as tetra- or penta-cyclic structures but some acyclic, mono-, bi-, tri- and hexa acyclic. As described in Table 4, Lupeol, lupine, botulin and oleanolic acid are some examples of pentacyclic triterpenoids (Furtado et al., 2017). Triterpenoids were detected from root and stem barks of *E. natalensis* (Khan, 1985). Phytol (0.66%) and squalene (5.85%) were detected from hexane extract of *E. crispa* using GC-MS (Palanisamy & Ashafa, 2018).

**TABLE 4:** Terpenoids isolated from Euclea species.

No	Name of the compound	Species	Plant part	Structure	References
**24**	20 (29)-lupene-3α-isoferulate	E. natalensis	RB	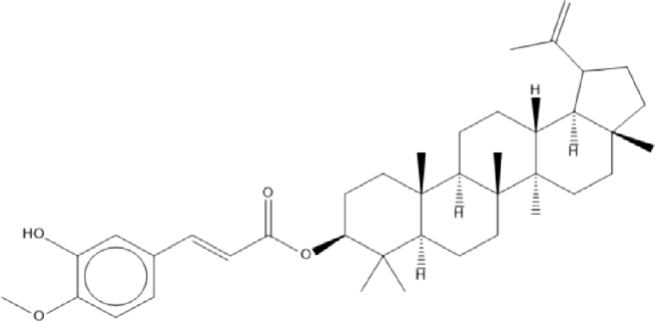 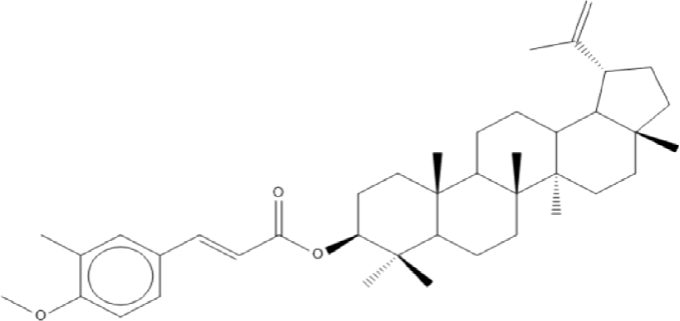	Weigenand et al. (2004)
**25**	20 (29)-lupene-3β-isoferulate	*E. natalensis*	RB	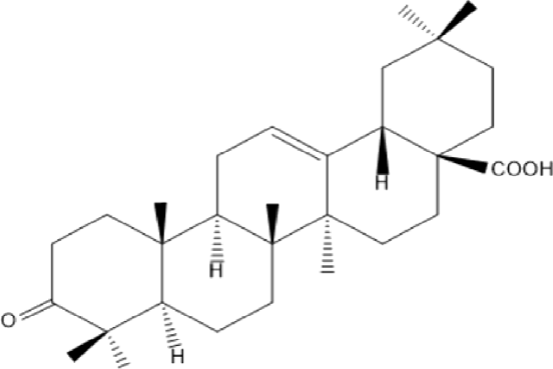 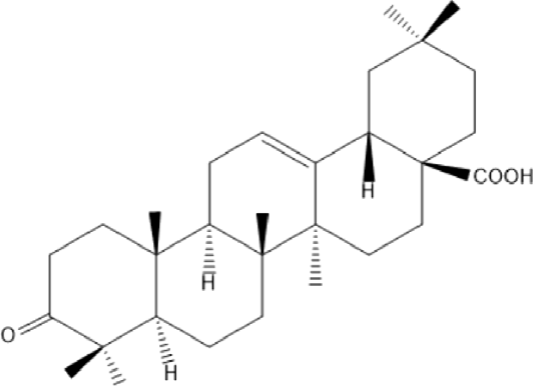	Lall et al. (2006)
**26**	3-oxo-oleanolic acid	E. crispa subsp. crispa	Us	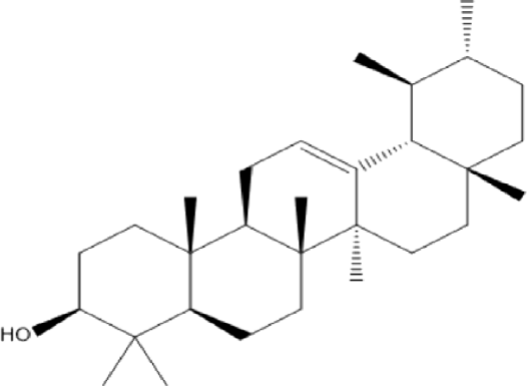	Kwon et al. (2011)
**27**	3β-(5-hydroxyferuloyl)lup-20 (30)-ene	E. crispa subsp. crispa	Us	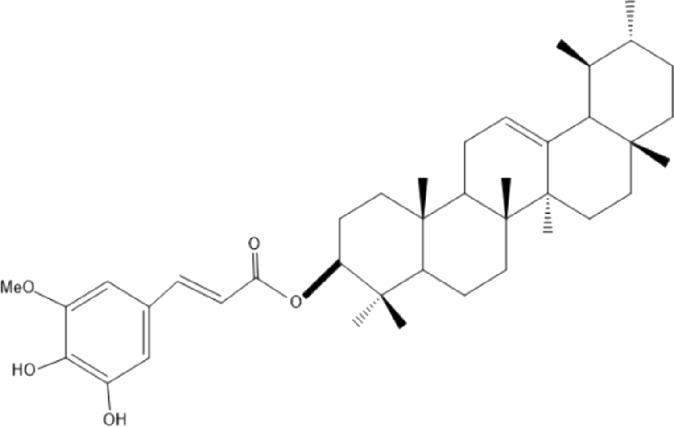	Kwon et al. (2011)
**28**	α-amyrin	*E. kellau*	L	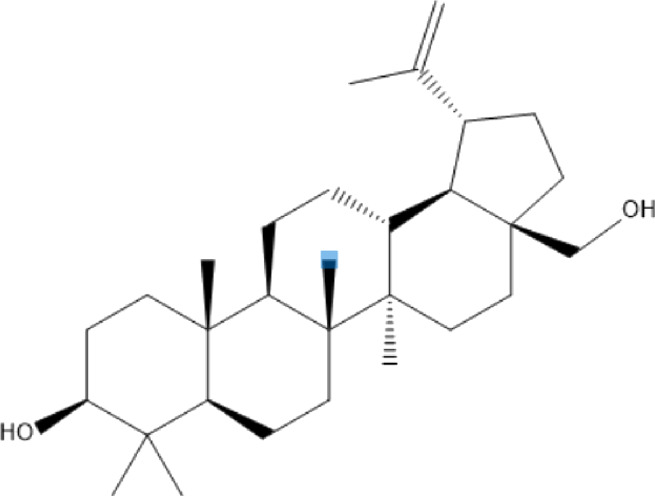	Orzalesi et al. (1970)
**29**	α-amyrin-3O-ß-(5-hydroxy) ferulic acid	*E. undulate*	RB	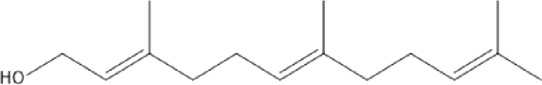	Deutschländer, 2010; Botha, 2016
**30**	betulin	E. natalensis	RB	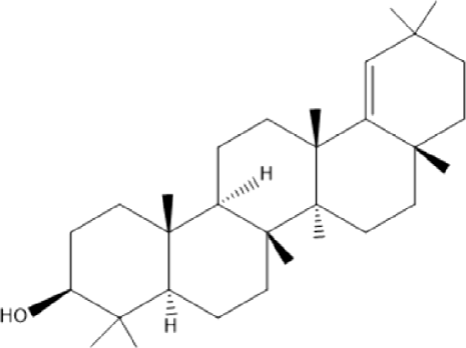 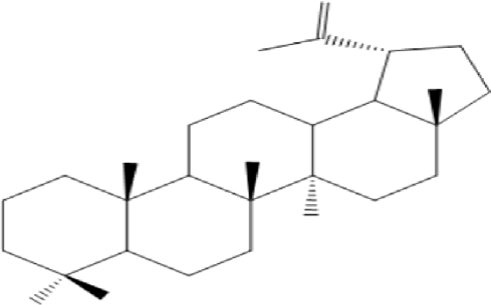	Weigenand et al., 2004; Lall et al., 2005b; Lall et al., 2006
		*E. divinorum*	RB		Mebe et al., 1998; Al-fatimi, 2019
		*E. kellau*	Br		Orzalesi et al. (1970)
		*E. latideus*	R		Philip et al. (2018)
		*E. undulata*	RB		Deutschländer, 2010
		*E. crispa* subsp. *crispa*	RB		Rademana et al. (2019)
**31**	farnesol	*E. crispa*	L	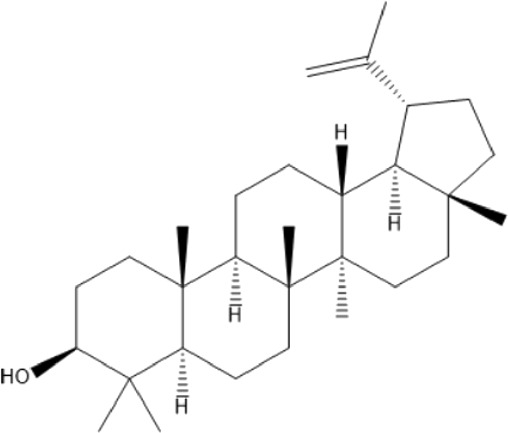 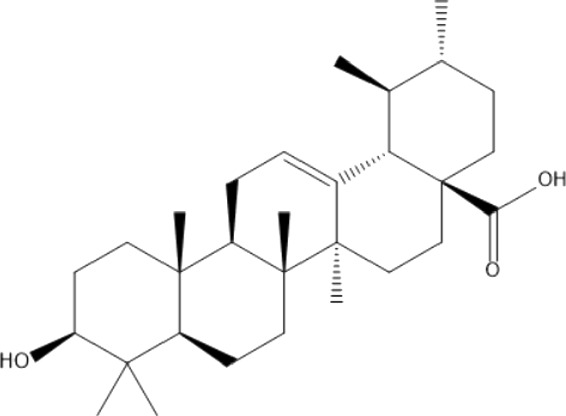	Palanisamy et al. (2020)
**32**	germanicol	*E. divinorum*	RB	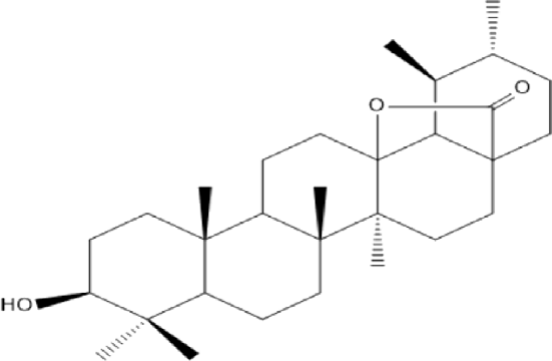 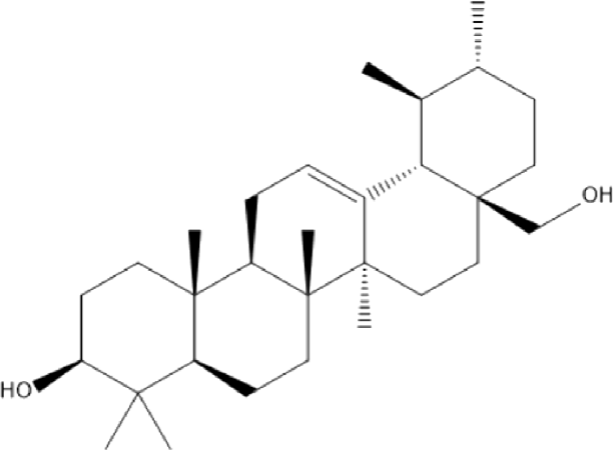	Kilonzo et al. (2019)
**33**	lupene	*E. divinorum*	RB	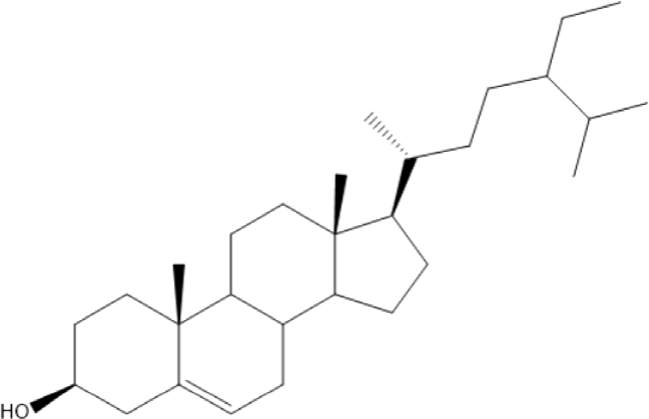 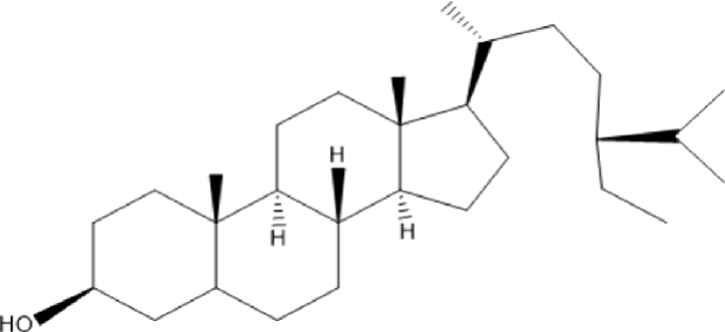	Mebe et al., 1998; Al-fatimi, 2019
**34**	lupeol	E. natalensis	RB	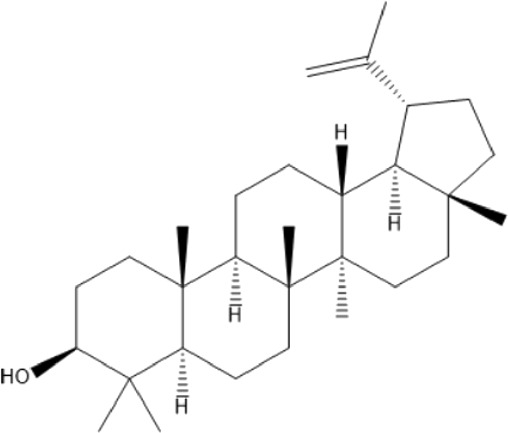 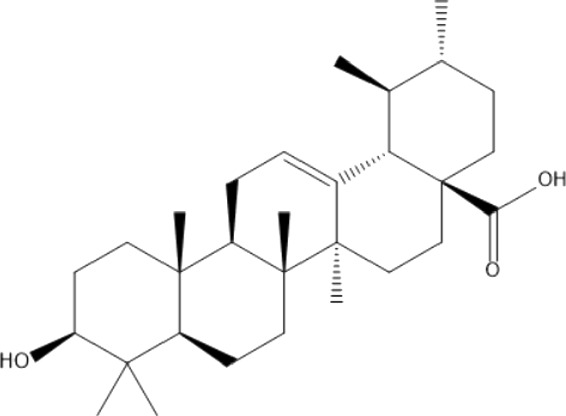	Weigenand et al., 2004; Lall et al., 2005a; Lall et al., 2006
		*E. divinorum*	RB		Mebe et al., 1998; Al-fatimi, 2019
		*E. kellau*	Br		Orzalesi et al. (1970)
		*E. latideus*	R		Philip et al. (2018)
		*E. undulata*	RB		Deutschländer, 2010; Botha, 2016
**35**	ursolic acid	*E. kellau*	Br, L	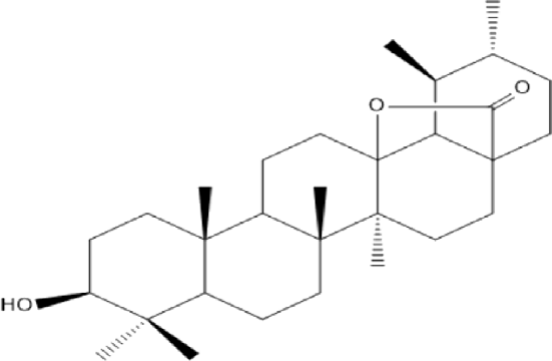 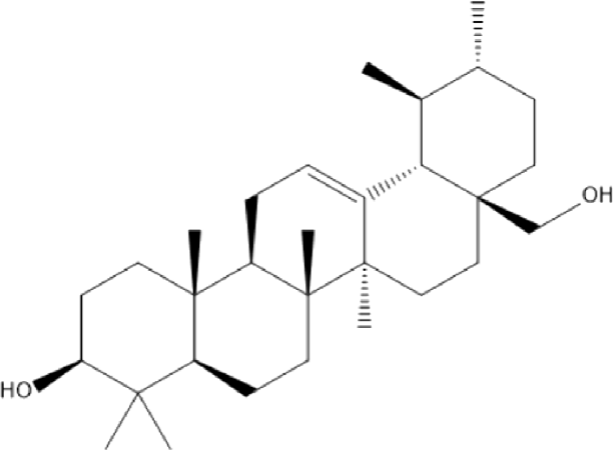	Orzalesi et al. (1970)
**36**	ursolic acid lactone	*E. kellau*	Br, L	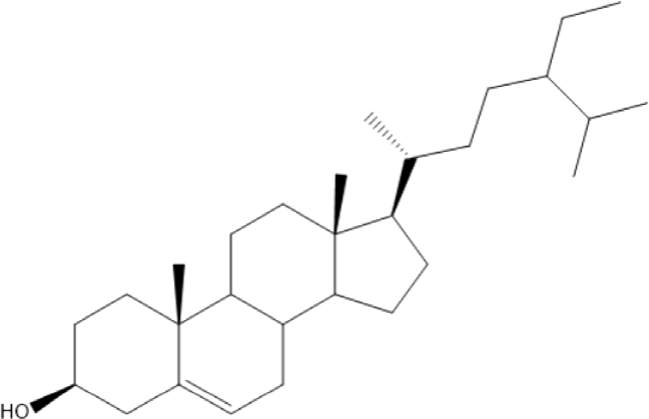	Orzalesi et al. (1970)
**37**	uvaol	*E. kellau*	L	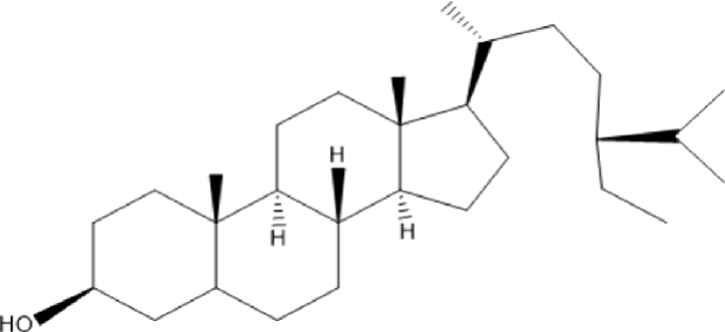	Orzalesi et al. (1970)
**38**	β-sitosterol	*E. natalensis*	RB	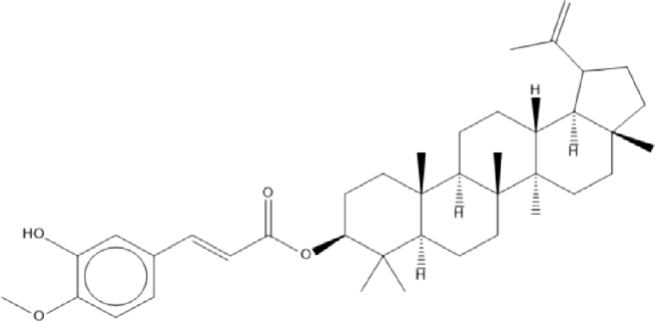	Lall et al., 2006; Moosavi et al., 2020
**39**	γ-sitosterol	*E. divinorum*	RB	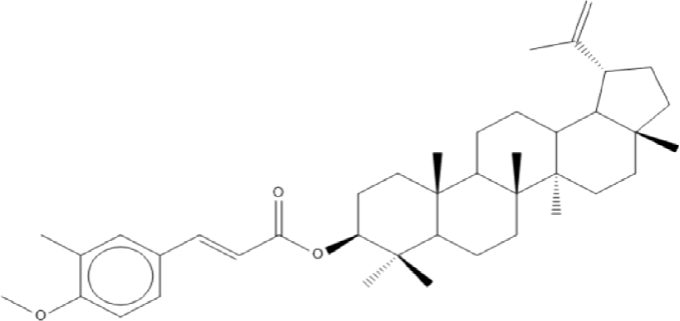	Kilonzo et al. (2019)

Br, Branch; L, leaf; R, root; RB, root bark; Us, Unspecified.

### Miscellaneous

The following bioactive compounds with their composition were identified from hexane extract of *E. crispa* using GC-MS: tetracosane (14.98%), dodecane (10.76%), 2-ethyl-1-decanol (8.00%), tridecane (7.53%), diphenyl vinyl phosphine (6.38%), triacontane (5.27%), 2,6-dimethylheptadec-ane (5.02%), docosane (3.68%), tetradecane (3.59%), 1-hepten-3-ol (2.63%), orthotolidine (2.31%), Phenyl glucuronide (2.25%), 5-tridecy-lbenzene-1,3-diol (1.90%), and Pentadec-ane (1.68%) (Palanisamy & Ashafa, 2018). Vitamin E, fatty acid methyl esters such as saturated (C_14_, C_20_) and unsaturated (C_16_, C_18:1_, C_18:2,_ and C_18:3_ were isolated from twigs and leaves of *E. undulate* (Maroyi, 2017). VTLC identified gallic and ellagic acid esters in E. *schimperi* (Mueller-harvey et al., 1987)*.*

### Biological activities

#### Antimicrobial activity

The acetone and aqueous extract of E. natalensis inhibited the growth of *Bacillus* cereus, B. pumilus, *B. subtilis*, *Micrococcus* kristinae, and *Staphylococcus aureus* at concentrations ranging between 0.1 and 6.0 mg/mL (Lall and Meyer, 2000). Isolated compounds from the root extract also demonstrated a significant antimicrobial effect. Diospyrin and 7-methyljuglone were more effective against Gram-positive bacteria than Gram-negative bacteria.

Shinanolone, 7-methyljuglone, diospyrin, isodiospyrin and neodiospyrin in the genus *Euclea* especially *E. natalensis* are potent for the treatment of both drug-sensitive and resistant tuberculosis (Joubert et al., 2006; Johanna, 2007; Bapela et al., 2008; Babula et al., 2009; Babiaka et al., 2015). On the other hand, diospyrin, lupeol, betulin and 7-methyl juglone presented in *E. natalensis* has inhibitory activity against drug-sensitive *M. tuberculosis* at MIC of 8.0 and 0.5 mg/mL respectively (Maroyi, 2017). The intracellular and extracellular inhibition of the latter compound is greater than that of the anti-tuberculosis drugs streptomycin and ethambutol (Lall et al., 2005b; Mcgaw et al., 2008).

7-methyl juglone and mamegakinone are effective against *M. tuberculosis* (Kooy et al., 2006), *Neisseria gonorrhoeae*, *Shigella dysenteriae* and *Shigella flexneri*. Aqueous and acetone extracts of the roots of *E. natalensis* inhibited the growth of *Mycobacterium tuberculosis* at MIC value of 0.5 mg/mL while MIC values for *B. cereus*, *B. pumilus*, *B. subtilis*, *M. kristinae* and *S. aureus* ranged from 0.1–6.0 mg/mL (Lall & Meyer, 2000; Lall and Meyer, 2001). 7-methyl juglone is also effective against *Saccharomyces cerevisiae*, *M. bovis*, *M. smegmatis* and *M. fortuitum* (Mbaveng & Kuete, 2014). Due to Shinanolone*, E. natalensis* inhibits the growth of Gram-positive bacterial strains and a drug-sensitive strain of *M. tuberculosis* at a concentration of 0.1 mg/mL (Weigenand et al., 2004).

Ethanolic extract of E. crispa leaves elicit antimicrobial activity with maximum inhibition zone against *Staphylococcus aureus, Streptococcus aureus, Escherichia coli, Klebsiella pneumonia, Aspergillus niger* and *Aspergillus terreus* (Palanisamy et al., 2019). Previous literatures demonstrated that E. lanceolata, E. undulata and E. multiflora possess antifungal activity due to the presence of lawsone, juglone and 7-methyljuglone (Lall & Meyer, 2000; Lall and Meyer, 2001). *Euclea natalensis* comprises β-sitosterol (Lall et al., 2006; Moosavi et al., 2020), 20 (29)-lupine-3β-isoferulic and shinanolone that have inhibitory activity against *Aspergillus niger* at 0.01 mg/mL. The former compound and octahydro euclein significantly show fungistatic activity against *C. cladosporioides* at 0.01 mg/mL. Besides this, octahydro euclein present in this plant is very effective for *Phytophthora* sp. at 0.1 mg/mL (Lall et al., 2006).

Ethyl acetate root extract of *E. divinorum* has inhibitory activity against Gram-negative bacteria like *E. coli* but is ineffective for *S. aureus.* Alkaloids and terpenoids in this plant contribute to this kind of antibacterial activity (Shumba, 2018). The MIC values of the extracts of *E. divinorum* against bacterial activity for root bark ethyl acetate and leaf aqueous ranges from 0.048-0.871 mg/mL and 0.781-1.562 mg/mL respectively. The first extract is very effective against *S. typhi* followed by stem bark aqueous and root bark petroleum ether extract against *S. aureus* (Kilonzo et al., 2019).

The non-polar dichloromethane root extract of *E. divinorum* root bark has better antifungal activity than the nystatin for *Absidia corymbifera, Aspergillus fumigatus, Candida krusei, Microsporum gypseum, Mucor sp*. and *Trichophyton mentagrophytes*. This activity is maintained with lupeol, lupine, botulin, 7-methyl juglone, diospyrin, iso diospyrin and shinalone (Al-fatimi, 2019).

#### Antiviral activity

The acetone extract of E. natalensis demonstrated moderate antiviral activity against HSV-1, at concentrations of 0.1–0.02 mg ml^−1^ (Lall et al., 2005a). In a study conducted by Tshikalange et al. (2007) 7-methyljuglone (potent), diospyrin, neodiospyrin, isodiospyrin, and 6-methyljuglone isolated from that E. natalensis exhibited HIV-1 reverse transcriptase activity at the concentrations ranging from 25 to 50 μg/mL. The leaf extract of *E. schimperi* showed good antiviral activity against Influenza A virus and herpes simplex virus (HSV-1) with IC_50_ values of 6.22 6 μg/mL and 67.5 μg/mL, respectively (Gebre-Mariam et al., 2006).

#### Antimalarial activity

Aqueous, dichloromethane, and methanol leaf and twig extracts of *E. undulata* have shown antimalarial activity against *Plasmodium falciparum* using the parasite lactate dehydrogenase assay (Maroyi, 2017). *E. latideus is* also effective against *P. falciparum* especially for the chloroquine resistant strain of *P. falciparum* due to the presence of lupeol, betulin, and 3β-(5-hydroxy feruloyl) lup-20 (30)-ene (Philip et al., 2018). The dichloromethane and methanol (1:1) root and leaf extracts of *E. natalensis* demonstrated promising activity in a research by Clarkson et al. (2004) employing the parasite lactate dehydrogenase assay, with (IC_50_) values of 5.1 and 5.3 mg/mL, respectively, against *P. falciparum*. A study done by Philip et al. (2018) indicated that the extracts and isolated compounds from *E. latideus* demonstrated antiplasmodial activity against chloroquine sensitive and chloroquine resistant strains of *P. falciparum.* The leaves of *E. natalensis* also showed antiplasmodial activity with an IC_50_ of 25.6 μg/mL (Tajuddeen et al., 2022). The *in vivo* antimalarial assay of the aqueous root extract of *E*. *divinorum* possessed significant parasitemia suppression (Girmaw and Engidawork, 2022).

#### Antidiabetic activity

*E. undulata* containing α-amyrin-3-O-β-(5-hydroxy) ferulic acid inhibits α-glucosidase and epicatechin lowers glucose levels in the blood (Botha, 2016). Phenolic acids and flavonoids of *E. crispa* inhibit alpha amylase with IC_50_ values of 1.001 mg/mL and 1.65 mg/mL (Tinevimbo, 2017). Lowering of blood glucose can be achieved with acetone root bark extracts of E. undulata by displaying a glucose uptake of 162.2% by changing liver cells at 50 mg/mL (Maroyi, 2017). *E. coriacea* contains phytosterols that possess antidiabetic activity (Mugomeri et al., 2014). Acetone root bark extracts of *E. undulata* effectively reduced fasting blood glucose levels, raised cholesterol, and triglyceride levels to close to normal without causing weight gain in an *in vivo* model of streptozotocin-nicotinamide-induced type-2 diabetes (Deutschländer et al., 2012).

#### Antioxidant activity

Ethanolic root bark and leaf extracts of E. crispa have radical scavenging activity because of flavonoids, phenolics (Tinevimbo, 2017) and (6E, 10E)-2, 6, 24-trimethylpentane cosa-2, 6, 10-triene isolated from the leaves of E. crispa exhibited potent antioxidant activity (Palanisamy et al., 2019). The leaves of *E. crispa* were tested for antioxidant activity and showed IC50 values of 113.79, 109.59, and 116.65 μg/mL for DPPH, hydroxyl and nitric oxide radical scavenging assays. Farnesol contributes to such activity (Palanisamy et al., 2020). At a 2000 mg/mL concentration, E. divinorum inhibits DPPH by 82.5%, 74.5% and 62.5% for the methanol fraction, aqueous fraction and crude extract, respectively (Feyissa et al., 2013). Fatty acids, flavonoids, and phenolics of *E. undulata* showed antioxidant activity using the DPPH, ABTS and FRAP assays (Maroyi, 2017). The free radical scavenging effect of methanol and chloroform leaf extracts of *E. schimperi* was demonstrated. The methanol and chloroform extracts were able to scavenge the DPPH radical with a percentage scavenging activity of 85.4% and 58.5% at the concentration of 40 μg/mL, respectively (Mekonnen et al., 2018).

#### Anticancer activity

The leaves of *E*. *crispa* subsp. *crispa* extract exhibited anti-proliferative activity on human breast adenocarcinoma (MCF-7) and human epidermoid carcinoma (A431) cell lines with IC_50_ values of 45.7 μg/mL and 41.8 μg/mL, respectively (Rademana et al., 2019). 7-methyl juglone and 3β-(5-hydroxy feruloyl) lup-20 (30)-ene, which are the main constituents of *E. divinorum*, showed anticancer effects against human breast cancer, colon cancer, fibrosarcoma, nasopharyngeal carcinoma, lung cancer, and human melanoma (Mebe et al., 1998). Diterpenes isolated from *E. coriacea* has been reported to possess an anticancer effect in human cells (Mugomeri et al., 2014). 7-Methyl juglone isolated from *E. racemosa* ssp. *schimperi* has been described to possess significant cytotoxic properties against human colon carcinoma cells (Wube et al., 2005). *Euclea natalensis* also contains this compound that has anticancer activity on several cancer cell lines, such as KB, Lu1, and LNCaP (Mbaveng & Kuete, 2014).

### Other activities

*E. coriacea* contains phytosterols that possess anti-inflammatory and anti-pain activity (Mugomeri et al., 2014). A study showed that *E. natalensis* shoot extract has *in vivo* hepatoprotective activity by reducing the level of alanine transaminase liver enzyme by 15% (50 mg/kg) and 40% (100 mg/kg). This plant also provides an immunomodulatory activity by increasing T-helper 1 cell cytokines such as Interleukin 2, Interleukin 12, and Interferon α by 12 fold and decreasing the T-helper 2 cell cytokine, interleukin 10 by 4 fold when compared to baseline cytokine production (Lall et al., 2016). The *in vivo* evaluation of the antidiuretic activity of *E. divinorum* revealed that the aqueous and methanol root extract of the plant possessed a significant diuretic activity by increasing urine volume and electrolyte excretion (Woldemedhin et al., 2017). Feyissa et al. (2013) demonstrated that the crude extract and solvent fractions of *E. divinorum* leaves restored gentamicin-induced nephrotoxicity by decreasing tubular necrosis, serum and oxidant markers and by increasing in antioxidant molecules. The methanol fraction provided the most renoprotection, implying that semi-polar antioxidant principles may be involved.

### Acute toxicity, gentotoxicity and cytotoxiciy

Acute toxicity studies of the crude and methanolic extract of *E. divinorum* leaves indicated that it was safe when administered orally at 2000 mg/kg (Feyissa et al., 2013; Woldemedhin et al., 2017). After a period of 72 h, the animals tolerated the administered dose, and there were no appreciable changes in behavior such as motor activity, diarrhoea, breathing, alertness, restlessness, convulsions, coma and appearance. Since no mortality was recorded within 14 days, the lethal dose (LD_50_) was indicated to be more than 2000 mg/kg. Shauli, (2023) evaluated the acute and sub-acute oral toxicity of *E. natalensis* and the results demonstrate that no treatment related deaths or toxic signs were observed. Another study done by Ayele et al. (2023) revealed that *E. racemosa* was safe after oral toxicity study with LD_50_ greater than 2000 mg/kg. *E. latideus* is considered as a non-toxic plant since acute toxicity studies showed that crude extracts had LD_50_ > 5,000 mg/kg (Kodi et al., 2018).

Taylor et al. (2003) investigated genotoxicity in human peripheral blood lymphocytes of South African medicinal plants. The results reported that the dichloromethane root extract of *E. divinorum* induced DNA damage (more cells with high tail DNA content), which was however lower than that of the positive control (1 mM potassium bichromate). However, the bark extract of *E. natalensis* showed positive results for genotoxicity in the micronucleus test.

## Conclusion

The genus *Euclea* is well known for its use in the treatment of diabetic and body pain manifestations. The traditional claims were justified by different biological evaluations. The genus *Euclea* is known to be a source of biologically active compounds. More than 40 compounds were isolated from the genus and naphthoquinones, pentacyclic triterpenes and flavonoids are the most abundant bioactive secondary metabolites which are responsible for the observed biological activity. Most of these secondary metabolites are found in the roots and root bark while some in fruit, seeds, leaves and shoots. According to the present review, it has been noted that the potential uses of the species in the treatment of viral infections and nerve-related diseases have not been scientifically explored. We believe the scientific community researching on the genus will benefit from the material compiled in this review.
